# Building a Client Resource and Communication Platform for Community-Based Organizations to Address Health and Social Needs: Co-Design Study

**DOI:** 10.2196/53939

**Published:** 2024-08-16

**Authors:** Courtney Lyles, Beth Berrean, Ana Buenaventura, Svetlana Milter, Dayana Daniel Hernandez, Urmimala Sarkar, Christian Gutierrez, Nynikka Palmer, William Brown III

**Affiliations:** 1 UC Davis Center for Healthcare Policy and Research, UC Davis School of Medicine, University of California, Davis, Sacramento, CA, United States; 2 Technology Services Unit, School of Medicine, University of California, San Francisco, San Francisco, CA, United States; 3 Department of General Internal Medicine, Zuckerberg San Francisco General Hospital, University of California, San Francisco, San Francisco, CA, United States; 4 Division of Prevention Science, Department of Medicine, University of California, San Francisco, San Francisco, CA, United States

**Keywords:** mHealth, mobile health, eHealth, electronic health, application, digital health, digital ecosystem, informatics, community-based, community, co-design, human-centered design, community health, population health, technology, innovation, operations, social needs, health resources, qualitative analysis

## Abstract

**Background:**

Connecting individuals to existing community resources is critical to addressing social needs and improving population health. While there is much ongoing informatics work embedding social needs screening and referrals into health care systems and their electronic health records, there has been less focus on the digital ecosystem and needs of community-based organizations (CBOs) providing or connecting individuals to these resources.

**Objective:**

We used human-centered design to develop a digital platform for CBOs, focused on identification of health and social resources and communication with their clients.

**Methods:**

Centered in the Develop phase of the design process, we conducted in-depth interviews in 2 phases with community-based organizational leadership and staff to create and iterate on the platform. We elicited and mapped participant feedback to theory-informed domains from the Technology Acceptance Model, such as Usefulness and Ease of Use, to build the final product and summarized all major design decisions as the platform development proceeded.

**Results:**

Overall, we completed 22 interviews with 18 community-based organizational leadership and staff in 2 consecutive Develop phases. After coding of the interview transcripts, there were 4 major themes related to usability, relevance, and external factors impacting use. Specifically, CBOs expressed an interest in a customer relationship management software to manage their client interactions and communications, and they needed specific additional features to address the scope of their everyday work, namely (1) digital and SMS text messaging communication with clients and (2) easy ways to identify relevant community resources based on diverse client needs and various program eligibility criteria. Finally, clear implementation needs emerged, such as digital training and support for staff using new platforms. The final platform, titled “Mapping to Enhance the Vitality of Engaged Neighborhoods (MAVEN),” was completed in the Salesforce environment in 2022, and it included features and functions directly mapped to the design process.

**Conclusions:**

Engaging community organizations in user-centered design of a health and social resource platform was essential to tapping into their deep expertise in serving local communities and neighborhoods. Design methods informed by behavioral theory can be similarly employed in other informatics research. Moving forward, much more work will be necessary to support the implementation of platforms specific to CBOs’ needs, especially given the resources, training, and customization needed in these settings.

## Introduction

Research supports expanding screening and referral for social needs within health care systems [1]. Approximately 20% of health outcomes are associated with medical care, while the remaining influences are social, community, and structural factors—sometimes referred to as social needs and the broader social determinants of health [2]. Health systems need to work collaboratively with community-based organizations (CBOs) to address unmet social needs that affect health and health care outcomes [3].

Much of the research to date has focused on health care system electronic health record (EHR)–based platforms that can send and receive information from community organizations about social needs resource referrals, such as new EHR screening tools to assess social needs domains like food or housing insecurity, as well as EHR-integrated platforms that can recommend and connect patients to relevant programs in the community. There is emerging evidence that these EHR-based platforms can improve screening and referrals, but downstream impacts on health care and health outcomes are less clear [4]. However, there is a need for better digital platforms explicitly *within* CBOs that identify or connect clients to social and community resources to promote health. Even though CBOs communicate with community members about health and social needs and connect clients with other community resources, they have much different workflows than clinicians and staff in health care settings and they do not have access to existing EHR platforms. Digital design focused outside of the health care system is essential to contributing to a future broader ecosystem of referrals and connections between community organizations, social services agencies, public health agencies, and health care settings [5].

We employed human-centered design methods to build a platform to support CBOs in understanding client social resource needs and connecting them with the most relevant resources in neighborhoods within San Francisco. We outline here our design methods and the concrete platform build decisions that emerged from our work with community organizations, as both the methodological approaches and the design features from this work are relevant for other organizations and projects focused on social and health needs.

## Methods

### Study Design

As a part of a 4-year National Institutes of Health project titled “Mapping to Enhance the Vitality of Engaged Neighborhoods (MAVEN),” we built and then pilot-tested a digital platform for CBOs serving communities within the San Francisco Bay Area. While this project focused on specific health and social resource referrals in San Francisco, the design methods as well as the final platform characteristics are relevant for many other communities working on the same questions and processes for CBOs. This work was informed by the Double Diamond design framework throughout all phases of the study [6]. We have previously published the Discovery and Define phases of this project, in which we conducted extensive qualitative work with community members and community leaders to identify the core audience and design principles for this work. In brief, in the Discovery phase, we highlighted CBOs as the pivotal audience for digital platform development (given their central role in resource provision and trusted roles within their communities) [7]. Then, the Define phase highlighted the need to build a broad and scalable digital platform to support resource needs in multiple domains and for diverse sets of clients or populations served [8]. In the next Develop phase of work outlined here, we focused on the final build of the MAVEN digital platform.

While not all design studies are theoretically oriented, we combined the rigorous design methods with a well-established theory to additionally bolster the rigor of the work and further enhance the generalizability of the study. In particular, we used the Technology Acceptance Model to frame our interview guides and analysis [9]. This theory summarizes core concepts of technology acceptance and use into major domains, such as Ease of Use and Usefulness, which have been validated in many previous studies assessing how and why technologies are adopted [9]. Thus, the addition of theory to our design work allowed us to ensure that participant feedback was consistently documented and categorized against validated constructs throughout the study.

### Building the MAVEN Platform: the Develop Phase

In the Develop phase, we completed 2 iterative rounds of design with CBOs in the San Francisco Bay Area (interview guide available in Multimedia Appendix 1). First, we completed 8 interviews with CBOs in late 2020 and early 2021 to understand their existing workflows using digital or online platforms to track and communicate with their clientele. At the end of these open-ended discussions, we also asked community leader participants to look at example digital resources to explore the features and relevance of these type of platforms in their everyday work. For example, community leaders reviewed websites with resources (such as on SF Department of Children, Youth and Their Families), providing overall opinions about usability as well as the types of information and content they might want to see displayed [10]. This phase was primarily open-ended and hypothetical, given that the platforms shown to participants were not always relevant for their own work. The interviews were conducted and recorded via Zoom (Zoom Video Communications, Inc), with the audio then converted into text via professional transcription.

After this round of feedback was completed, we created a minimal viable product (MVP) of the MAVEN social resource tool for CBOs and conducted additional co-design interviews. An MVP is a standard step in the design processes in which the team creates the simplest working prototype that can be tested, which is critical before more intensive digital building [11]. The MVP was created in spring 2021, and we completed an additional 13 think aloud interviews with CBO leaders to test the MVP in May and June 2021 [12]. These video interviews were conducted and recorded via Zoom, and the audio was then professionally transcribed. The think aloud process asked each participant to navigate the MVP on their own sequentially through each task or feature in the platform (eg, logging in, adding clients, sending a communication to a client), with prompts at every task that elicited technical errors or roadblocks as well as participants’ overall opinions and reactions to the MVP.

### Data Analysis

We used qualitative descriptive methods to complete our analyses within the Develop phase of the work, combining the participant feedback across all iterations of the platform build [13]. The text of transcribed interviews was analyzed using Dedoose (SocioCultural Research Associates) qualitative analysis software, and the second-round interview transcripts were also compared side by side with videos of the MVP think aloud procedures. Using open coding, we first identified overall patterns of feedback from participants, holding regular team meetings to establish consensus on the major usability and content feedback categories that emerged (ie, specific feature needs and preferences, technical barriers to use). Next, we mapped these categories onto Technology Acceptance Model domains to examine how the overall discussion categories were related to documented theoretical constructs such as relevance or usefulness as well as ease of use of the platform.

Finally, we made overall design decisions informed by these qualitative findings to incorporate into the final MAVEN platform. More specifically, we used the data from both rounds of feedback to prioritize new functionality that needed to be added to the platform, as well as improvements or changes in the MVP features that would improve relevance or usability. These design changes are summarized here, with the final MAVEN tool completed in the spring of 2022.

### Ethical Considerations

This study was approved by the University of California San Francisco (institutional review board no. 18‐25696). The participants provided written consent for the interviews to be audio and video recorded, and the transcripts were deidentified prior to the analysis. Each participant was compensated US $75 per interview.

## Results

### Study Sample

We completed 22 in-depth interviews (9 in the first phase and 13 in the second phase of the study), with 18 unique CBO leaders and staff in total (4 participants were interviewed in both phases of work). The 18 participants in the Develop phase of the MAVEN project work ranged from individual consultants working on multiple health campaigns in their local neighborhoods to mid-sized nonprofit organizations running programs and provisioning services in their communities. The full summary of participants is shown in Table 1.

**Table 1.** Summary of community-based organizations staff or leader participants.

Phase	Organization type	Participant title or role	Location
1	Nonprofit	Director of community partnerships and program evaluation for HIV programs	San Francisco, CA
1	Nonprofit	Agent and disability resource specialist	Mission Neighborhood, San Francisco, CA
1	Nonprofit	Information and assistance specialist	Mission Neighborhood, San Francisco, CA
1	Nonprofit	Program director, women’s cancer program	Tenderloin Neighborhood,San Francisco, CA
1	Nonprofit	Program coordinator, HIV/AIDS programs	San Francisco, CA
1, 2	Community advocate	Health educator and activist	San Francisco Bay Area, CALos Angeles, CA
1, 2	Nonprofit	Senior services manager	Mission neighborhood, San Francisco, CA
1, 2	Local planning council;nonprofit	Member; senior director of programs	Tenderloin neighborhood,San Francisco, CA
1, 2	Nonprofit	Executive director	San Francisco, CA
2	Local government housing or community organization	Director, social/health resources	Tenderloin neighborhood,San Francisco, CA
2	Regional advisory group; local government organization	Advisory member; HIV case manager	Alaska, Hawaii, California
2	Nonprofit	Program director, senior services programming	San Francisco, CA
2	Local government housing or community organization	Program director, digital programming	Tenderloin Neighborhood,San Francisco, CA
2	Community advocate	Community health/HIV activist	San Francisco, CA
2	Local governmentPublic health department	Community health outreach/HIV activist	Alameda, CA
2	Federally qualified health center	Health educator	Marin City, CA
2	Nonprofit	Wellness manager	Bay View Neighborhood,San Francisco, CA
2	Public research university system	Health equity strategist/consultant	San Francisco, CA

### Qualitative Results

Analyzing all in-depth interviews during the Develop phase, we identified 4 major themes from community leader participants, which are mapped to the Technology Acceptance Model domains of Usefulness, Ease of Use, and External Factors influencing use.

### Overall Usefulness

In the domain of usefulness, there was consensus that access to technology platforms with the ability to track clients across programs and communicate with clients more seamlessly was a priority for CBOs. Several of the organizations’ current workflows involved using out-of-the-box and free programs for their daily work of tracking clients, finding and referring people to resources, and communicating with clients. While existing platforms allow digital communication with clients and tracking client contact information or program participation, the standard work was often nonintegrated and potentially duplicative. For example, one participant stated:

*If you want to refer somebody or some participant to other services,…you can Google it and just find out the exact address and telephone that they can call. I think it’s the best tool that we can have now to just give more information and referrals.*

It was clear from these participants that better functionality beyond free platforms would improve their daily work at CBOs, such as by standardizing the search and communication workflows into a single place.

More specifically, there was an understanding among community leaders that existing consumer or customer resource management (CRM) platforms, such as Salesforce (Salesforce, Inc) and other similar CRM software, were important to be able to complete multiple client functions together—such as tracking clients and communicating with clients (individually or in groups) without toggling between multiple different platforms. For example, one participant stated:

*I love [seeing]…a lot of information and a lot of resources at the same time. So, I don’t have to look for another screen and start looking for another information for the clients*.

Thus, any CRM platform that combined database management for clients alongside communication tools was mentioned as more ideal for CBO workflows. Specific to the selection of a CRM software, participants most often mentioned Salesforce as the platform of interest (either currently in use or a wish for use in the near future): “I think at least 50% [of organizations we partner with] are using some portion of Salesforce [with] their clients.”

Relatedly, the usefulness of up-to-date lists of community-based resources was a top priority for almost all participants. For example, one community leader stated, “I think that [technology] would be a good way to maybe have a resource list of frequently used CBOs [and resources they provide].” Similarly, another participant stated:

*I think being able to see what [resource] is available—and I think if this stuff is updated in real time, it’s super helpful…. Having specific contacts [at each organization] for different opportunities[/resources] is really key just because I think that’s often something that people spend a lot of time trying to determine*.

However, it was clear that this registry of community resources would require upkeep and trust in the resources presented. For example, a participant mentioned:

*[A registry of] existing community resources—that’s a lot to keep up-to-date. And if you just don’t have it, then you’re not disappointing people [clients]*.

A final useful function of a platform for CBOs centered around communication with clients. Comments such as these solidified the need to have integrated and customizable communication with clients as a core function of the platform: “You…have subgroups of different clients depending on what the [program] is, and then you would just create a new [communication] for whatever thing you were doing,” and “I feel like, just knowing colleagues at other places, a lot of people are paying money for text and email blasts.”

### Overall Ease of Use

Next, there were a number of usability issues that emerged during the interviews.

In addition, when interacting with the early MVP of the MAVEN platform, there were several usability comments made about the layout and the terminology, especially in the context of community-oriented work. One participant stated it as:

*I just want to make sure that [you know that] some of these words that you’re using are a little technical…. Maybe finding other words…that will describe something that’s more simple and easier to find*.

And another participant mentioned:

*Since it’s our first time, it’s going to be difficult just to find everything, but once you get to know it, I think just it’s going to take less than a week just to see and working with the database or with the website*.

While the overall usability ratings of the MVP were favorable, there were several areas of improvements that community leaders mentioned that were specific to increasing the usability of a platform that served diverse clients and were necessary in many CBOs’ workflows. First, participants identified a need to have easier ways to find recommended resources for clients, as exemplified in comments such as:

*[Make it] easy to find if it’s in alphabetical order or has some kind of a search engine that I could put in ‘Help with income taxes’ or ‘Grocery shopping’ or whatever the [client] needs*.

Similarly, another participant stated that it is important to ensure that referrals to resources match their participants’ eligibility and identity:

*[To make sure] you could search by eligibility requirement, whether it’s senior citizen, or HIV-positive, or somebody who identifies as LGBTQ*.

Next, participants identified clear, equity-focused improvements to the platform, especially around language accessibility and sociodemographic representation of fields (particularly careful collection of sexual orientation and gender identity data for LGBTQ+ populations). For example, one participant stated, “When I talk about cultural sensitivity, like I said, it’s not just a language also. If it’s from LGBTQIA community or different cultures, it’s very, very important. Age also is another issue,” and another stated, “I know that many agencies have different [languages], for example, Chinese and English, also, Russian and English, Spanish and English…. So, it should be in multiple languages.”

### External Factor: Varying Implementation Contexts Within CBOs

Finally, there were many external factors that emerged as critical to use of the MAVEN platform. These comments generally focused on the implementation context for using such a digital platform.

First, there was a clear need to focus on digital skills and necessary training among CBO staff. For example, participants stated, “We know Salesforce has really high potential, but we don’t have the time to learn it all…. So, we don’t use this fancy thing because it was just too overwhelming to use, to learn it and so we’re just comfortable with what we know,” and “I feel like there’s certainly a spectrum of computer literacy across my team, and so I think getting folks that are less technologically adept onboard for using something like this could be a little bit challenging, but I don’t think it would be that hard.”

Participants also discussed that no platform would be successful without leadership support as well as very detailed training among frontline staff. For example, participants stated, “I feel like it’s important to have a collective buy-in or an agreement or some kind of commitment to using a certain resource to sort of get it off the ground,” and “Oftentimes, there are lots of tools that you will get sent that will be given to somebody but I think they’re only as good as the people that are using them.” Furthermore, there were common comments about on-the-ground support for staff members at CBOs such as, “I was thinking more training for community leaders in how to really effectively use this tool.”

In addition, there were more nuanced conversations with CBOs about their current workflows with clients that needed to be considered in order to get uptake of a new system. In particular, because CBO staff used in-person and phone outreach often for clients, any digital tool would require consideration of how to blend such high-touch, face-to-face communication with the lower-touch messaging and reminders that digital platforms can facilitate. Quotes such as these exemplified this idea:

*So it’s about having a conversation with the client and understanding how they best connect the services and also what barriers may be in the past they have faced when trying to connect with other providers and sort of strategizing ways to circumvent those challenges*.

Comments from CBOs about their workflows highlighted that even a perfect digital solution that easily facilitated messaging may supplement but not replace high-touch conversations with clients about their preferences for resources.

Finally, some of the conversation about implementation centered around 2 additional points that related to the overall uptake and dissemination of any platform. The security and privacy of information in any client database was critical, especially given the sensitive information disclosed by clients and the known predatory behavior of hackers and other criminal behavior. This was exemplified in the following quote: “Organizations may take advantage of…elderly patients.” Moreover, the implementation considerations for wider spread and uptake of digital platforms for health and social needs also centered on the practical work of maintenance, which cannot be overlooked. For example, one participant stated:

*There is a lot of movement that occurs in public service organizations that having the most up to date data or contacts could be a concern and a lot of work on us to continue to update*.

In other words, the ability to use existing resource lists that are actively updated and monitored for relevance is essential for implementation and will not be solved by a technical solution alone.

### Final Design Decisions and Build

Given the qualitative findings across both the open-ended and MVP testing of the MAVEN platform, we made multiple design decisions that reflected the community-based organizational leadership input and preferences. Overall, we chose to design within Salesforce, activate several features within the platform, and then integrate outside tools to provide information and tools within the platform. Table 2 summarized core findings matched to concrete features or changes within the MAVEN platform.

**Table 2.** Major design needs from co-design feedback.

Design needs identified by participants	Subsequent feature or design decision
*Platform need*: Community leaders used or wanted CRM^a^ platform to manage their clients and workflows.	Selection of Salesforce platform for MAVEN^b^ build.
*Feature need*: Community leaders had knowledge of social or health resources but strongly desired a trusted and easy-to-use directory to improve client resource referrals.	Review of several options for an up-to-date list of community resources; selection of San Francisco Service Guide existing resource list (imported into MAVEN via API^c^).
*Feature need*: Simple and seamless communication was a priority to reach and stay connected to clients.	Activation of Twilio within Salesforce Campaigns to allow for 2-way texting functionality.
*Feature need*: Matching of available resources to specific client needs and identities was critical.	Selection of ServiceMatch feature from Salesforce, which uses an algorithm to recommend “best” resource by resource type or category (eg, food), zip code, and eligibility criteria (eg, language of service provision).
*Usability improvement*: Improvement of language and data collection fields within digital platforms needed to better represent client identities.	While using existing CRM platform, we expanded client intake fields to be better representative of the community; exploration of designing in multiple languages (but unable to do so in this Develop period).
*Implementation consideration*: Workflows and staffing capacity within CBOs^d^ varied greatly and needed to be prioritized.	Implementation support and training identified as a high priority during rollout.

a CRM: customer resource management.

b MAVEN: Mapping to Enhance the Vitality of Engaged Neighborhoods.

c API: application programming interface.

d CBO: community-based organization.

**Figure 1.**
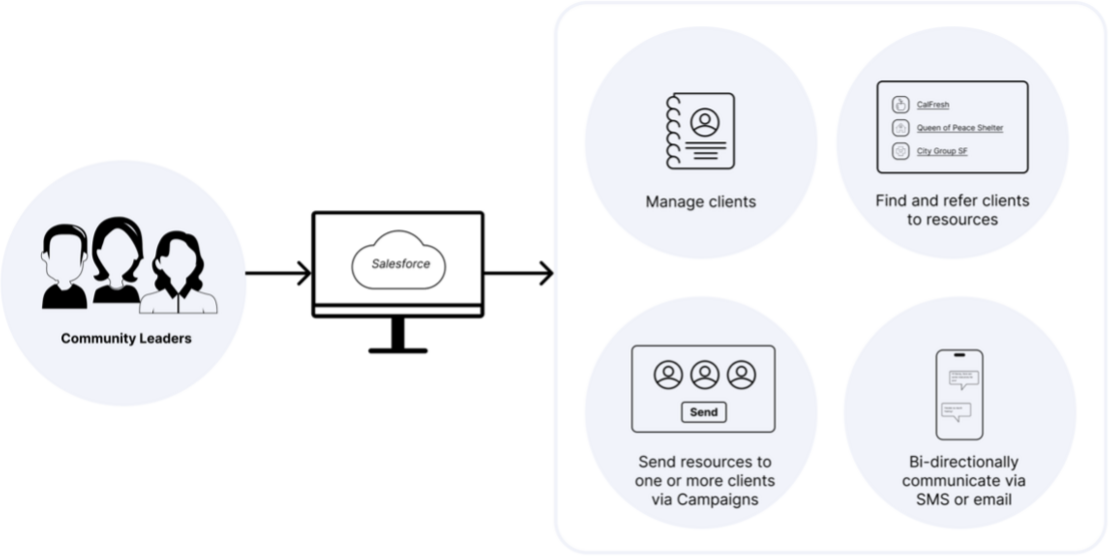
MAVEN minimal viable product. MAVEN: Mapping to Enhance the Vitality of Engaged Neighborhoods; SF: San Francisco.

We ended up implementing these design decisions in stages within our work. In the first MVP build of the MAVEN platform (Figure 1), we identified the core platform and communication functionality needed:

1. Salesforce environment, as this was a platform used in about half of existing community organizations to track client and program information (but only accessible or viewable within their own organization). This selection was regularly expressed as a desired platform by most CBOs in the study.
2. Enabling the Campaign feature within the Salesforce environment, which allows group SMS and email communication within the platform (on top of existing client-tracking features). Thus, community leaders could login, create new contacts or clients, and also create new messages with any content they developed or inserted.
3. Adding Twilio-for-Salesforce–managed package to seamlessly allow 2-way SMS text messaging via the platform.

**Figure 2.**
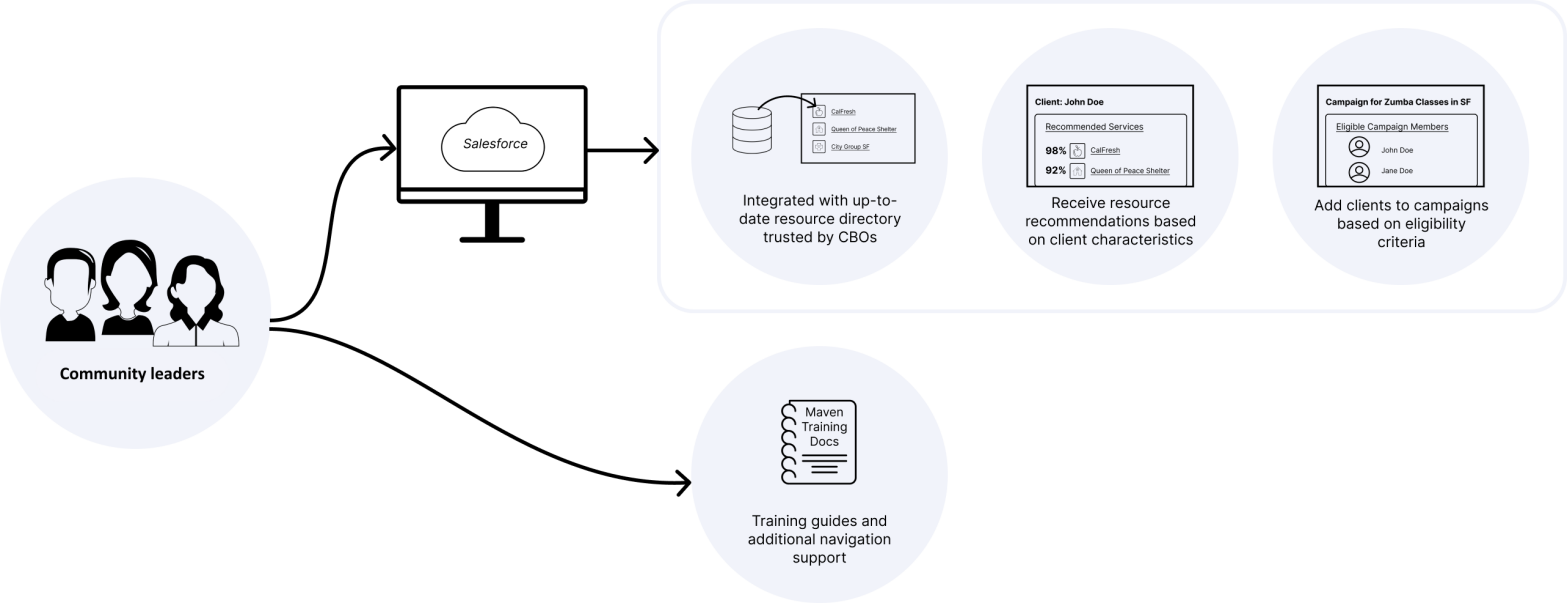
MAVEN phase 2. CBO: community-based organization; MAVEN: Mapping to Enhance the Vitality of Engaged Neighborhoods; SF: San Francisco.

After the second phase of testing an MVP of the MAVEN platform, we identified additional platform features as new final components of the tool (Figure 2):

1. Integration with an existing resource directory that was trusted among community organizations. After review of several existing lists of community resources in San Francisco, we chose a freely available and regularly updated resource list called the “San Francisco Service Guide” (managed by a nonprofit called “ShelterTech”) [14]. This registry of resources included categories of resources: locations, hours, contact information for each resource, and some free-text eligibility information for the resource (eg, available to those aged 65 years or older via a senior center) [14]. We connected SF Service Guide via application programming interface into Salesforce and built an automated refresh process to update the list of records on a weekly basis. The underlying SF Service Guide repository was regularly updated every 90 days by volunteers at ShelterTech.
2. Ability to receive “recommendations” for a client resource based on category of resource, location, and eligibility criteria. We chose a freely available and open-source tool within the Salesforce environment called ServiceMatch, which was available via Salesforce.org Impact Labs [15]. We customized the existing code for ServiceMatch within MAVEN, adjusting the algorithm to be able to use the existing resource list information to make recommendations of the most relevant resources based on client characteristics (eg, eligibility of resource based on age, location [ie, client zip code], and resource type [ie, search for food vs housing]). Finally, connecting ServiceMatch with Twilio allows community leaders to refer these relevant resources to clients via SMS in addition to email and printout with 1 click.
3. Extension of existing Campaigns Salesforce tool that automatically adds clients to the campaign based on the resource eligibility criteria and allows referral of multiple resources to 1 or more clients. Thus, the community leaders can create a campaign and add multiple resources to the campaign, and the system automatically selects clients based on the unified eligibility criteria across all campaign resources selected. Broadcast messaging service was used to send bulk SMS messages to clients included in the campaign.
4. Rollout of platform was completed with the addition of implementation support. Finally, we created detailed user guides and training materials to support CBOs in using a platform that was often very new to their daily workflow and addressed some of the major navigation questions that were confusing in the platform.

The final MAVEN product combined elements from multiple places that did not previously exist: the Campaigns feature (with additional communication functionality), plus curated resources directly from SF Resource Guide, plus searching of resources by type, location, and eligibility via ServiceMatch.

## Discussion

### Principal Findings

This paper outlines a co-design process to create and iterate a digital platform for CBOs, focused on finding relevant health and social resources and easily connecting clients to these resources. Overall, human-centered design methods elucidated core needs and preferences of CBOs for health and social resource screening and referrals. This is an audience that has often been overlooked in the clinical informatics literature given that CBOs work outside of the EHR and have entirely different workflows with their clients as compared with a health care environment [16,17]. Specifically, we outlined multiple platform features and design decisions that matched the needs expressed during in-depth qualitative interviews, applying a rigorous and theory-informed approach to the design.

Our findings are likely to have relevance outside of the San Francisco context because of the design choices made in this study. First, many CBOs across the country are looking to use CRM software and specifically Salesforce to manage client communication, which was also reflected in our study findings. Therefore, there is the ability for other groups to use the existing Salesforce functionality identified in the MAVEN study (such as ServiceMatch and the Campaigns feature with Twilio integration). In addition, we chose to integrate an existing resource list via application programming interface into the MAVEN platform, which can be swapped out for any other resource list based on the local context in other communities. Importantly, the co-design process in this study to build the MAVEN platform led not only to the enhancement of the tool but also to specific attention and focus on the context for using the tool, such as use of existing operating platforms, workflow considerations, staffing and workforce capacity, and flexibility of the tool to meet the diverse needs of clients served [18,19].

Moreover, this study is a useful example of the Develop phase of the design work, which emphasizes real-world implementation and potential constraints, which are a critical complement to the open brainstorming and ideation of the Discovery phase of design [20]. Moving forward in this field, it will also be critical for future studies to focus on human-centered design to advance health equity. Equity-focused design methods are spreading, and these methods are essential for building platforms that prioritize the needs of marginalized communities and address multiple levels of influences on health outcomes [21,22].

### Limitations

This study had several limitations, such as the completion of the work in a single geographic area (the San Francisco Bay Area) and the relatively small number of organizations participating in the design. However, both the design methods and the findings that highlight implementation considerations are widely generalizable to many other cities and municipalities (in addition to Salesforce build considerations). Finally, the budget of this research project also limited the number of phases of iterations and the final set of features activated. Similarly, because of budget, we were not able to transition use of the MAVEN platform from the University of California San Francisco (UCSF) Salesforce license (where the study testing occurred) to independent licenses at CBOs, which remains a barrier for longer-term uptake.

### Conclusions

Moving forward, there is a strong need for more community-engaged research and informatics co-design to support the development and implementation of platforms that assist CBOs in their daily work. Understanding individual social needs within health care delivery systems is essential for health promotion, but solutions that require delivering health and social resources within local neighborhoods will not be solved by health care systems alone. In order to partner with CBOs and follow their expertise, we also cannot ignore the resources, capacity, and skills or training needs in these organizations (as our study and many others have highlighted), as real impact will be diminished without investment in CBOs based on their priorities.

## Supplementary material

10.2196/53939 Multimedia Appendix 1 Semistructured interview questions for community-based organizational leaders.

## Abbreviations

CBO: community-based organization
CRM: customer resource management
EHR: electronic health record
MAVEN: Mapping to Enhance the Vitality of Engaged Neighborhoods
MVP: minimal viable product
