# Supplementary material for: Building a Client Resource and Communication Platform for Community-Based Organizations to Address Health and Social Needs: Co-Design Study
Source: JMIR Hum Factors. 2024 Aug 16;11:e53939. doi: 10.2196/53939 (PMC11342060; doi:10.2196/53939)
Supplement: Multimedia Appendix 1 [file humanfactors-v11-e53939-s001.docx]

**Multimedia Appendix 1.**

*Could you start off telling us a little bit about you and the role your organization plays?*

*Tell us a little bit about the technology you use in your day-to-day work with community members...*

1. What are you mandated to use? Which would you continue to use if you weren’t required to do so?
2. What kinds of screening or additional data do you track about your community members?
3. What tracking systems do you have that you use for internal reporting or program development?
4. What do you like most about the tech tools you have right now?
5. What are your biggest challenges using technology in your everyday work, especially when working with clients?
   1. PROBE: communicating with clients both inside the office and “on the go”
6. Who in your organization owns decisions about which technology system you use? Do they also own the implementation of this solution?
7. How often do clients come in? How do you typically communicate with your clients?

## **Access/Install Salesforce Website/App**

Share Salesforce tool link and username and password with participant. Complete log-in and then have community leader share screen.

## **Specific Usability Tasks**

1. Look at the Contacts feature and then practice adding a new participant to the contacts list
2. Review the different resources available
   1. Pick one resource, or add a resource you typically refer your clients to
      1. *Click on New*
   2. Try and send this resource to the following client: [[Pick a client]]
      1. Would you prefer to email or SMS? Complete communication as preferred
3. Campaigns
   1. Try sending an email campaign with this resource
   2. Send a bulk SMS to a group of clients with another resource linked

## **Follow-Up Semi-Structured Questions**

1. What are your initial thoughts after downloading/reviewing how to use this tool?
   1. What was challenging about this process?
   2. What do you think worked well?
   3. What would you change?
2. What kind of scenarios do you anticipate using this tool?
3. Any concerns you already have about the tool?
4. What types of resources are you typically referring clients to?
5. How do you anticipate using the tool with your clients?
6. What are you interested in testing or using more?
7. Anything else that you think we should know before we continue further developing a tool like this?
